# Outcomes and factors influencing survival in cirrhotic cases with spontaneous rupture of hepatocellular carcinoma: a multicenter study

**DOI:** 10.1186/1471-230X-9-29

**Published:** 2009-04-30

**Authors:** Hiroyuki Kirikoshi, Satoru Saito, Masato Yoneda, Koji Fujita, Hironori Mawatari, Takashi Uchiyama, Takuma Higurashi, Kento Imajo, Takashi Sakaguchi, Kazuhiro Atsukawa, Aya Sawabe, Akira Kanesaki, Hirokazu Takahashi, Yasunobu Abe, Masahiko Inamori, Noritoshi Kobayashi, Kensuke Kubota, Norio Ueno, Atsushi Nakajima

**Affiliations:** 1 Gastroenterology Division, Yokohama City University Graduate School of Medicine, Yokohama, Japan; 2 Gastroenterology Division, Hiratsuka City Hospital, Hiratsuka, Japan; 3 Gastroenterology Division, Machida Municipal Hospital, Machida, Japan

## Abstract

**Background:**

Spontaneous rupture is rare complication of hepatocellular carcinoma (HCC) with high mortality rate in cirrhotic cases. The aim of this study was to determine the factors influencing prognosis in cases of spontaneously ruptured HCC and to investigate the outcomes of the treatments employed, especially transcatheter arterial embolization (TAE).

**Methods:**

A retrospective multicenter study was conducted in 48 cirrhotic patients with spontaneous rupture of HCC. Conservative treatment was employed in 32 patients (ConT group) and TAE was performed in 16 patients (TAE group).

**Results:**

The median survival time (MST) in the ConT group was only 13.1 days and the survival rate was extremely poor: 59.4% at 7 days, 37.5% at 14 days, and 6.3% at 30 days. On the other hand, the MST in the TAE group was 244.8 days and the survival rate was 87.5% at 1 month, 56.3% at 3 months, 23.4% at 12 months, and 15.6% at 24 months. According to the results of univariate analyses, factors associated with poor hepatic function and poor suitability for TAE was important determinants of short-term death (less than 3 weeks) among the patients (*p *< 0.05). On the other hand, among the patients in whom initial TAE was successfully performed (*n *= 15), a multivariate analysis showed that a maximum tumor size not exceeding 7 cm was the only independent factor determining long-term survival (*p *= 0.0130).

**Conclusion:**

Despite the inherent limitations of this retrospective study, TAE appears to be a useful treatment strategy for cirrhotic patients with spontaneous HCC rupture, as it yielded a longer survival period compared with conservative treatment in patients with ruptured HCC. Among the patients with ruptured HCC in whom initial TAE was successfully performed, the maximum tumor size was an important factor influencing survival.

## Background

Hepatocellular carcinoma (HCC) is one of the most common neoplasms encountered, and its incidence is increasing worldwide because of the increasing prevalence of hepatitis C virus infections [1-5]. One of the life-threatening complications of HCC is the spontaneous rupture of the tumor, with intra-peritoneal hemorrhage. Spontaneous rupture of HCC occurs in 3 to 15% of all patients with HCC, and high mortality rates in the range of 32 to 100% have been reported [6-15]. The following treatments have been employed for the treatment of ruptured HCC: emergent hepatic resection, placation or packing, hepatic artery ligation, and transcatheter arterial embolization (TAE) [9-18]. However, patients with advanced HCC tend to have poor hepatic function as a result of chronic hepatitis or liver cirrhosis. Therefore, a better understanding of the disease, wider recognition of this potential complication, improved methods of assessing liver function preoperatively, and less invasive methods of hemostasis may contribute to improved survival in cases of ruptured HCC.

The aim of this study was to determine the factors associated with prognosis in cases of spontaneously ruptured HCC and to investigate the outcomes of treatment, especially transcatheter arterial embolization (TAE).

## Methods

From January 1995 to May 2008, a total of 48 cirrhotic patients with spontaneously ruptured HCC were admitted to the Yokohama City University Hospital (33 patients), the Hiratsuka City Hospital (10 patients), and the Machida Municipal Hospital (5 patients).

The diagnosis of HCC was made based on the identification of a space-occupying lesion(s) in the liver using computed tomography (CT) or ultrasonography (US). The presence of hemoperitoneum in the patients was diagnosed by the demonstration of bloody fluid in the peritoneum by an abdominal puncture. Spontaneous rupture was the initial symptom of HCC in 4 patients, while in the remaining 44 patients, the spontaneous rupture of HCC was diagnosed during the follow-up period for liver cirrhosis (LC) or HCC.

The past medical history, vital signs, blood counts, blood chemistry, coagulation test results, levels of tumor markers, progression profile of HCC (number of tumors, maximum tumor size, presence of portal vein tumor thrombosis and presence of extra-hepatic metastasis), treatments employed, and survival rates were recorded. Shock at the time of admission was defined as a systolic blood pressure of < 80 mmHg and pulse rate of > 100 b.p.m. The LC status was evaluated using the Child-Pugh criteria using a combination of clinical (presence of hepatic encephalopathy, ascites) and laboratory data (serum bilirubin levels, prothrombin time (PT) and serum albumin levels). Staging of the HCC was performed according to the TNM staging system proposed by the Liver Cancer Study Group of Japan [19]: stage I (fulfillment of three intra-hepatic conditions: solitary, no more than 2 cm, no vessel invasion), stage II (fulfillment of two out of three intra-hepatic conditions), stage III (fulfillment of one of three intra-hepatic conditions), stage IVA (fulfillment of none of the three intra-hepatic conditions with no distant metastases, or any intra-hepatic condition with lymph node metastases), and stage IVB (any intra-hepatic condition with distant metastases). Patients were not included if they fulfilled one or more of the following criteria: presence of poorly controlled hepatic encephalopathy; presence of poorly controlled ascites; presence of jaundice (or a serum total bilirubin level higher than 5.0 mg/dL); or a poor performance status. An attempt was made to obtain informed consent for performing emergent TAE from all the patients, and the decision to perform TAE was made according to each patient's wishes. Emergent TAE was performed for hemostasis or the prevention of re-bleeding in 16 patients who provided their informed consent (TAE group, *n *= 16), while conservative treatment was employed in the remaining 32 patients (ConT group, *n *= 32). The patient characteristics in each group are shown in Table 1. This study was approved by the Ethics Committee of Yokohama City University School of Medicine.

**Table 1:** Comparison of patient characteristics between ConT and TAE groups

	Total	ConT group	TAE group	*p *– value
Patients (n)	48	32	16	
Age	69.3 ± 8.1	70.2 ± 8.7	67.5 ± 6.6	NS
Sex (n, %)				
Male	36 (75%)	22 (69%)	14 (87%)	NS
Female	12 (25%)	10 (31%)	2 (13%)	
Etiology of LC (n, %)				
HCV	32 (67%)	21 (65%)	11 (69%)	
HBV	4 (8%)	3 (9%)	1 (6%)	NS
Alcohol	5 (10%)	4 (13%)	1 (6%)	
NonB-nonC	7 (15%)	4 (13%)	3 (19%)	
Past history of HCC treatment (n, %)				
presence	36 (75%)	24 (75%)	12 (75%)	NS
absence	12 (25%)	8 (25%)	4 (25%)	
Admission duration for the treatment of HCC	3.0 ± 2.8	3.2 ± 2.9	2.6 ± 2.7	NS
Months from first treatment of HCC	31.0 ± 32.7	35.2 ± 36.4	22.8 ± 22.3	NS
Shock (n, %)				
presence	22 (48%)	16 (50%)	6 (38%)	NS
absence	26 (52%)	16 (50%)	10 (62%)	
Albumin (g/dL)	3.2 ± 0.4	3.1 ± 0.5	3.3 ± 0.3	NS
Total bilirubin (mg/dL)	1.8 ± 0.9	1.9 ± 0.9	1.5 ± 0.7	NS
PT (INR)	1.25 ± 0.13	1.24 ± 0.12	1.29 ± 0.16	NS
ICG at 15 min	33.1 ± 13.2	32.9 ± 12.9	33.5 ± 14.2	NS
AFP (ng/mL)	59163	84009	9472	NS
PIVKA-II (mAU/mL)	39589	55199	8370	NS
Ascites (n, %)				
presence	32 (67%)	23 (72%)	9 (56%)	NS
absence	16 (33%)	9 (28%)	7 (44%)	
Hepatic encephalopathy (n, %)				
presence	25 (52%)	17 (53%)	8 (50%)	NS
absence	23 (48%)	15 (47%)	8 (50%)	
AST (IU/L)	91 ± 73	94 ± 79	76 ± 51	NS
ALT (IU/L)	53 ± 35	52 ± 35	53 ± 35	NS
Hemoglobin (g/dL)	11.5 ± 1.9	11.1 ± 1.9	12.2 ± 1.8	NS
Platelet count (× 10^4^)	14.9 ± 7.8	14.1 ± 6.0	16.6 ± 10.5	NS
Creatinine (mg/dL)	1.03 ± 0.66	1.08 ± 0.75	0.92 ± 0.42	NS
Child-Pugh classification (n, %)				
A	12 (24%)	7 (22%)	5 (31%)	
B	18 (38%)	11 (34%)	7 (44%)	NS
C	18 (38%)	14 (44%)	4 (25%)	
Child-Pugh score	8.0 ± 2.1	8.2 ± 2.1	7.8 ± 1.9	NS
Number of tumors (n, %)				
single	6 (13%)	2 (6%)	4 (25%)	NS
multiple	42 (87%)	30 (94%)	12 (75%)	
Maximum tumor size (cm)	7.0 ± 2.0	7.1 ± 3.9	6.9 ± 2.8	NS
Maximum tumor size (n, %)				
≤ 7 cm	23 (48%)	15 (47%)	8 (50%)	NS
> 7 cm	25 (52%)	17 (53%)	8 (50%)	
Portal vein tumor thrombosis				
presence	24 (50%)	16 (50%)	8 (50%)	NS
absence	24 (50%)	16 (50%)	8 (50%)	
Clinical stage				
II	1 (4%)	0 (0%)	1 (6%)	
III	22 (44%)	12 (38%)	10 (63%)	NS
IVA	15 (31%)	11 (34%)	4 (25%)	
IVB	10 (21%)	9 (28%)	1 (6%)	

Abbreviations: ConT, conservative treatment: TAE, transcatheter arterial embolization: LC, liver cirrhosis: HCV, hepatitis C virus: HBV, hepatitis B virus: HCC, hepatocellular carcinoma: PT (INR), prothrombin time (international ratio): ICG, indocyanine green: AFP, alpha-fetoprotein: PIVKA-II, protein induced vitamin K absence-II: AST, aspartate aminotransferase: ALT, alanine aminotransferase: NS, not significant.

### Statistical analysis

The statistical analysis was conducted using StatView version 5.0 software (SAS, Cary, NC). Group comparisons were performed using the chi-square test for independence or the Fisher exact test for comparisons of the two groups. The overall survival rate in each group was determined using the Kaplan-Meier method and a log-rank test, whit the survival period defined as the length of time from the onset of the spontaneous rupture of the HCC until the death of the patient. A multivariate analysis was performed using the Cox hazard regression model. The cut-off level for each parameter was decided based on the median level for all the patients. P values < 0.05 were considered statistically significant. The closing date for the study was May 31, 2008.

## Results

The initial symptoms of spontaneous rupture of HCC were the sudden onset of abdominal pain (28 patients, 58%), shock at admission (22 patients, 46%), and abdominal distension (20 patients, 42%). With regard to the stage of LC, 12 patients were classified as Child-Pugh A (24%), 18 as Child-Pugh B (38%), and 18 as Child-Pugh C (38%). As for the stage of HCC, one patient was classified as clinical stage II (2%), 22 patients as stage III (46%), 15 as stage IVA (31%), and 10 as stage IVB (21%).

The HCC was located on the liver surface in all the patients. Most of the patients had advanced HCC, and 42 patients had multiple HCCs (88%); among these patients, 25 patients had a maximum tumor size of > 7 cm (52%), and 24 patients had portal vein tumor thrombosis (50%) (Table 1). Univariate analyses identified shock at admission (*p *= 0.0205), the presence of hepatic encephalopathy (*p *= 0.0431), a high Child-Pugh score (*p *= 0.0074), a high serum bilirubin level (*p *= 0.0052), a high serum aspartate transferase (AST) level (*p *= 0.0142), a low serum albumin level (*p *= 0.0008), and non-suitability for TAE (*p *< 0.0001) as determinants of a poor 21-day (50% survival time of all the patients) survival (Table 2). A multivariate analysis using the Cox hazard regression model (including all the patients; *n *= 48) identified TAE as the only independent factor determining a relatively long survival period (*p *< 0.0001) (Table 3).

**Table 2:** Univariate analysis of factors influencing overall 21-day survival (*n *= 48).

50% Survival time: 21 days	Patients who died within 21 days	Patients who survived longer than 21 days	*p *– value
Patients (n)	24	24	
Age	70.8 ± 8.2	67.8 ± 7.9	NS
Sex (n, %)			
Male	19 (79%)	17 (71%)	NS
Female	5 (21%)	7 (29%)	
Etiology of LC (n, %)			
HCV	16 (67%)	16 (67%)	
HBV	3 (13%)	1 (4%)	NS
Alcohol	3 (13%)	2 (7%)	
NonB-nonC	2 (7%)	5 (22%)	
Past history of HCC treatment (n, %)			
presence	19 (79%)	17 (71%)	NS
absence	5 (21%)	7 (29%)	
Admission duration for the treatment of HCC	3.4 ± 3.0	2.6 ± 2.7	NS
Months from first treatment of HCC	31.6 ± 28.0	30.5 ± 37.4	NS
Shock (n, %)			
presence	15 (62%)	7 (29%)	*p *= 0.0205
absence	9 (38%)	17 (71%)	
Albumin (g/dL)	3.0 ± 0.4	3.4 ± 0.4	*p *= 0.0008
Total bilirubin (mg/dL)	2.2 ± 1.0	1.5 ± 0.6	*p *= 0.0052
PT (INR)	1.24 ± 0.12	1.27 ± 0.15	NS
ICG at 15 min	35.6 ± 12.1	30.6 ± 14.0	NS
AFP (ng/mL)	92400	25926	NS
PIVKA-II (mAU/mL)	35513	43666	NS
Ascites (n, %)			
presence	12 (50%)	13 (54%)	NS
absence	12 (50%)	11 (46%)	
Hepatic encephalopathy (n, %)			
presence	16 (67%)	9 (38%)	*p *= 0.0431
absence	8 (33%)	15 (62%)	
AST (IU/L)	127 ± 86	76 ± 45	*p *= 0.0142
ALT (IU/L)	61 ± 42	44 ± 23	NS
Hemoglobin (g/dL)	11.1 ± 2.1	11.8 ± 1.7	NS
Platelet count (× 10^4^)	12.9 ± 5.5	16.9 ± 9.2	NS
Creatinine (mg/dL)	0.99 ± 0.40	1.06 ± 0.85	NS
Child-Pugh classification (n, %)			
A	2 (8%)	10 (42%)	
B	10 (42%)	8 (33%)	*p *= 0.0229
C	12 (50%)	6 (25%)	
Child-Pugh score	9.2 ± 1.8	7.6 ± 2.0	*p *= 0.0074
Number of tumors (n, %)			
single	1 (4%)	5 (21%)	NS
multiple	23 (96%)	19 (79%)	
Maximum tumor size (cm)	7.2 ± 3.5	7.7 ± 3.5	NS
Maximum tumor size (n, %)			
≤ 7 cm	13 (54%)	9 (38%)	NS
> 7 cm	11 (46%)	15 (62%)	
Portal vein tumor thrombosis			
presence	12 (50%)	12 (50%)	NS
absence	12 (50%)	12 (50%)	
Clinical stage			
II	0 (0%)	1 (4%)	
III	11 (46%)	11 (46%)	NS
IVA	8 (33%)	7 (29%)	
IVB	5 (21%)	5 (21%)	
Performing TAE			
Performed	1 (4%)	15 (62%)	*p *< 0.0001
Not performed	23 (96%)	9 (38%)	

Abbreviations: LC, liver cirrhosis: HCV, hepatitis C virus: HBV, hepatitis B virus: HCC, hepatocellular carcinoma: PT (INR), prothrombin time (international ratio): ICG, indocyanine green: AFP, alpha-fetoprotein: PIVKA-II, protein induced vitamin K absence-II: AST, aspartate aminotransferase: ALT, alanine aminotransferase: TAE, transcatheter arterial embolization: NS, not significant.

**Table 3:** Multivariate analysis of factors influencing overall survival (*n *= 48).

Factor	Odds Ratio	95% C.I.	*p *– value
Shock: presence	1.522	0.788 – 2.941	NS
Albumin > 3.2 g/dL	0.979	0.408 – 2.350	NS
Total bilirubin > 2.0 mg/dL	1.046	0.623 – 1.754	NS
Hepatic encephalopathy: presence	1.094	0.513 – 2.331	NS
AST > 90 IU/L	1.035	0.472 – 2.270	NS
Child-Pugh: C	1.858	0.686 – 5.031	NS
TAE performed	0.032	0.006 – 0.163	*p *< 0.0001

Abbreviations: AST, aspartate aminotransferase: TAE, transcatheter arterial embolization: NS, not significant.

**Figure 1:**
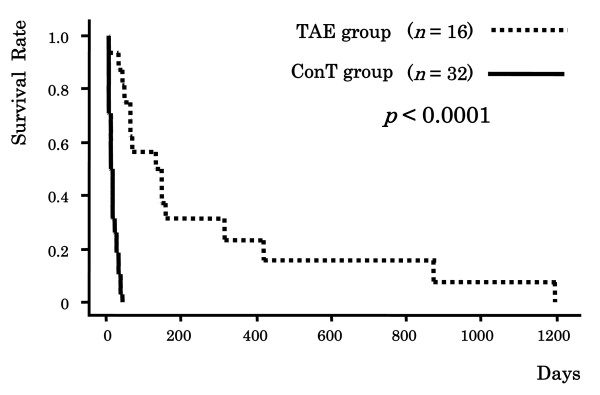
**Cumulative survival rates in the ConT (conservative treatment) group and in the TAE (transcatheter arterial embolization) group**.

TAE was performed in 16 patients. The extravasation of the blood from the ruptured HCC was revealed by digital subtraction angiography (DSA) in 9 patients (56%). TAE was successful in controlling acute hemorrhage in 15 patients (success rate, 94%). The MST in the TAE group was significantly better than that in the ConT group (244.8 days [range, 1–1200 days] vs. 13.1 days [range, 1–35 days], *p *= 0.0003). The cumulative survival rates in the ConT group were 59.4% at 7 days, 37.5% at 14 days, and 6.3% at 30 days. The cumulative survival rates in the TAE group were significantly superior to those in the ConT group, namely, 87.5% at 1 month, 56.3% at 3 months, 31.3% at 6 months, 23.4% at 12 months, and 15.6% at 24 months (*p *< 0.0001) (Figure 1).

In a further analysis of the patients in whom the initial TAE was successfully performed (*n *= 15), a univariate analysis identified a maximum tumor size exceeding 7 cm (*p *= 0.0052) as a significant determinant of a poor 140-day (median survival time of this group) survival, while the presence of portal vein tumor thrombosis (*p *= 0.0572) exhibited a tendency toward being a determinant factor (Table 4). A multivariate analysis identified only a maximum tumor size exceeding 7 cm (*p *= 0.0135) as an inverse independent factor determining relatively long-term survival (Table 5).

**Table 4:** Univariate analysis of factors influencing 140-day survival among patients in whom initial TAE was successfully performed (*n *= 15).

50% Survival time: 140 days	Patients who died within 140 days	Patients who survived longer than 140 days	*p *– value
Patients (n)	9	6	
Age (years)	67.1 ± 6.1	68.8 ± 8.2	NS
Sex (n, %)			
Male	8 (89%)	5 (83%)	NS
Female	1 (11%)	1 (17%)	
Etiology of LC (n, %)			
HCV	5 (56%)	5 (83%)	
HBV	1 (11%)	0 (0%)	NS
Alcohol	1 (11%)	0 (0%)	
NonB-NonC	2 (22%)	1 (17%)	
Past history of HCC treatment (n, %)			
presence	8 (89%)	4 (67%)	NS
absence	1 (11%)	2 (33%)	
Admission duration for treatment of HCC	3.6 ± 3.1	1.7 ± 1.6	NS
Months from first treatment of HCC	22.6 ± 21.9	27.0 ± 24.6	NS
Shock (n, %)			
presence	3 (33%)	3 (50%)	NS
absence	6 (67%)	3 (50%)	
Albumin (g/dL)	3.3 ± 0.4	3.4 ± 0.3	NS
Total bilirubin (mg/dL)	1.5 ± 0.7	1.6 ± 0.6	NS
PT (INR)	1.30 ± 0.19	1.29 ± 0.15	NS
ICG at 15 min (%)	35.1 ± 17.1	32.0 ± 11.0	NS
AFP (ng/ml)	1686	250	NS
PIVKA-II (mAU/mL)	7186	7758	NS
Ascites (n, %)			
presence	5 (56%)	4 (67%)	NS
absence	4 (44%)	2 (33%)	
Hepatic encephalopathy (n, %)			
presence	4 (44%)	4 (67%)	NS
absence	5 (56%)	2 (33%)	
AST (IU/L)	86 ± 59	66 ± 41	NS
ALT (IU/L)	58 ± 39	46 ± 33	NS
Hemoglobin (g/dL)	12.7 ± 1.4	11.7 ± 2.4	NS
Platelet count (× 10^4^)	16.7 ± 12.3	16.4 ± 9.5	NS
Creatinine (mg/dL)	0.80 ± 0.14	0.86 ± 0.16	NS
Child-Pugh classification (n, %)			
A	3 (33%)	2 (33%)	
B	4 (45%)	2 (33%)	NS
C	2 (22%)	2 (33%)	
Child-Pugh score	7.7 ± 2.0	8.0 ± 2.1	NS
Number of tumors (n, %)			
single	2 (22%)	1 (17%)	NS
multiple	7 (78%)	5 (83%)	
Maximum tumor size (cm)	7.4 ± 1.7	6.3 ± 3.3	NS (*p *= 0.1221)
Maximum tumor size (n, %)			
≤ 7 cm	1 (11%)	5 (83%)	*p *= 0.0052
> 7 cm	8 (89%)	1 (17%)	
Portal vein tumor thrombosis			
presence	6 (67%)	1 (17%)	NS (*p *= 0.0572)
absence	3 (33%)	5 (83%)	
Clinical stage			
II, III	5 (56%)	5 (83%)	NS
IVA, IVB	4 (44%)	1 (17%)	

Abbreviations: TAE, transcatheter arterial embolization: LC, liver cirrhosis: HCV, hepatitis C virus: HBV, hepatitis B virus: HCC, hepatocellular carcinoma: PT (INR), prothrombin time (international ratio): ICG, indocyanine green: AFP, alpha-fetoprotein: PIVKA-II, protein induced vitamin K absence-II: AST, aspartate aminotransferase: ALT, alanine aminotransferase: NS, not significant.

**Table 5:** Multivariate analysis of factors influencing the survival of patients in whom initial TAE was successfully performed (*n *= 15).

Factor	Odds Ratio	95% C.I.	*p *– value
Maximum tumor size > 7 cm	26.304	1.966 – 352.005	*p *= 0.0135
Portal vein tumor thrombosis: absence	0.444	0.080 – 2.475	NS (*p *= 0.3545)

Abbreviations: TAE, transcatheter arterial embolization: NS, not significant.

**Figure 2:**
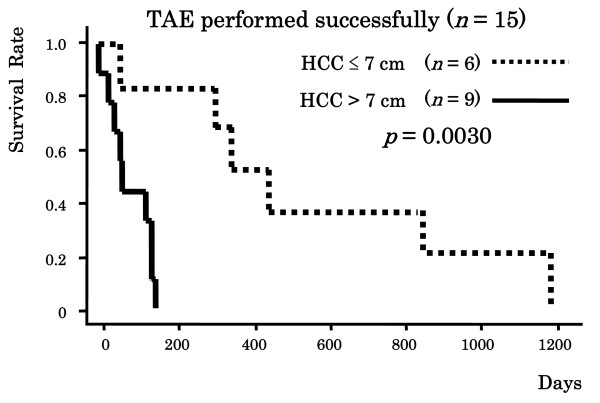
**Cumulative survival rates in patients according to maximum tumor size**. Abbreviations: TAE, transcatheter arterial embolization: HCC, hepatocellular carcinoma.

Among the patients in whom initial TAE was successfully performed, the MST among the patients in whom the maximum tumor size was ≤ 7 cm (*n *= 6) was significantly better than that among the patients in whom the maximum tumor size was > 7 cm (*n *= 9) (523.3 days [range, 60–1200 days] vs. 82.1 days [range, 1–150 days], *p *= 0.0080). Furthermore, the cumulative survival rate among the patients in whom the maximum tumor size was ≤ 7 cm was significantly better than that among the patients in whom the maximum tumor size was > 7 cm (100%, 83.3%, 83.3% and 50.0% vs. 77.8%, 44.4%, 0% and 0% at 1 month, 3 months, 6 months and 12 months, respectively; *p *= 0.0030) (Figure 2).

All the patients in the ConT group died within 35 days of hospitalization during the first hospital admission. In the ConT group, the main cause of death was re-rupture or re-bleeding of HCC (23 patients; 72%). On the other hand, in the TAE group, 9 patients (60%) were discharged from the hospital. Fifteen patients in the TAE group died during the follow-up period of this study. The main cause of death in the TAE group was hepatic failure (10 patients, 67%). Only two patients (13%) died of re-rupture or re-bleeding of HCC (Table 6).

**Table 6:** Causes of death in both groups

Treatment	No. of patients who died	Re-rupture or re-bleeding (%)	Hepatic failure (%)	Others (%)
ConT group	32	23 (72)	9 (28)	0 (0)
TAE group	15	2 (13)	10 (67)	3 (20)

In one of the 16 TAE patients, it was not possible to stop the hemorrhage by use of TAE.

Abbreviations: ConT, conservative treatment: TAE, transcatheter arterial embolization

## Discussion

Spontaneous rupture of HCC is a fatal complication of advanced HCC. While previous studies have shown a very poor prognosis, with a 30-day mortality rate in the range of 30 – 70% [10-15,20,21], recent reports have indicated a significant decrease in the mortality rate. In our study, we observed an overall 30-day mortality rate of 67%, while the mortality rate was only 6.7% (one patient) among the patients in whom initial TAE treatment was successful (15 patients).

Univariate analyses identified various factors, including the presence of hepatic encephalopathy, a high Child-Pugh score, a high serum bilirubin level, a high serum AST level, and a low serum albumin level (all of which are associated with poor liver function) as significantly influencing the short-term death rate (within 3 weeks) among the cases with ruptured HCC. In contrast, factors associated with the progression of HCC, such as the number of tumors, the maximum tumor size, and portal vein tumor thrombosis, were not revealed by univariate analyses to be significant factors influencing the short-term death rate in patients with ruptured HCC. A multivariate analysis including all the patients (*n *= 48) identified the performance of TAE as the only independent factor influencing survival. Regardless of the status of HCC progression, re-rupture or re-bleeding of HCC as well as short-term death could be prevented among patients in whom initial TAE was successfully performed. These results suggest that TAE is a useful treatment strategy for the spontaneous rupture of HCC and that factors reflecting the background liver function are important factors influencing short-term mortality.

Hepatic resection is one of the best treatment options for ruptured HCC, but it is technically difficult to perform in cirrhotic liver and its success depends on the severity of the cirrhosis. One group reported that only 12.5% of patients with ruptured HCC could be managed with hepatic resection, while another group reported a percentage of 59.3%. A similar situation was noted in relation to TAE treatment. Some groups reported the presence of shock at admission as an important factor influencing the survival time [14,16,20-22]. Shock in patients with the spontaneous rupture of HCC is caused by hypovolemia resulting from hemorrhage. In addition, patients with LC or liver failure have underlying coagulopathy. Therefore, emergent TAE is difficult to perform in patients with shock. In our study, a univariate analysis in the ConT group also identified shock at admission as an independent factor influencing the short-term death rate. Another group also reported that a serum total bilirubin level of higher than 2.92 mg/dL (50 μmol/L) was a critical predictor of survival. In a series of patients treated with TAE and reported by Ngan et al. [23], none of the patients with a serum total bilirubin level higher than 2.92 mg/dL (50 μmol/L) survived longer than 9 weeks (median survival, 1 week), while patients with a serum total bilirubin level of 2.92 mg/dL (50 μmol/L) or lower survived for 15 weeks, on average. In a series of patients treated with TAE and reported by Leung et al. [24], the mean survival of the patients with a serum total bilirubin level higher than 2.92 mg/dL (50 μmol/L) was only 34 days, while that of patients with a serum total bilirubin level lower than 2.92 mg/dL (50 μmol/L) was 165 days. In our study, which yielded similar results, the mean serum bilirubin level of the patients who died within 3 weeks was significantly higher than that of the patients who survived for more than 1 month (2.20 mg/dL vs. 1.51 mg/dL, *p *= 0.0052).

Patients with severe ruptures of HCC and poor liver function have an extremely guarded prognosis and cannot tolerate surgical hepatic resection. Therefore, in this study, TAE was aggressively pursued for the treatment of patients with ruptured HCC, and the results of a multivariate analysis revealed that the performance of TAE alone was a significant independent factor influencing the overall long-term survival. The MST in the TAE group was significantly longer than that in the ConT group. All the patients in the ConT group died within 35 days as a result of re-rupture, re-bleeding or hepatic failure. In contrast, the success rate of TAE was relatively high in our study.

Lau et al. [25] reported that the mean survival period of patients with ruptured HCC who had been treated with TAE was 218.3 days for Child-Pugh A patients, 83.4 days for Child-Pugh B patients, and 11.0 days for Child-Pugh C patients. However, in their study, the status of HCC progression was not described in detail. We consider that the growth of HCC plays an important role in the events leading up to rupture. In our study, a multivariate analysis revealed the size of the tumor to be the only independent factor influencing long-term survival among patients in whom initial TAE was successfully performed, and both the survival rate and the MST, as assessed using the Kaplan-Meier method, among the patients in whom the maximum tumor size was ≤ 7 cm were significantly better than those among the patients in whom the maximum tumor size was > 7 cm. Regarding the mechanism of rupture, Zhu et al. [26] reported that rupture might be initiated by the invasion and occlusion of the hepatic veins by tumor cells, resulting in an increase in pressure within the tumor. Such venous congestion would lead to central tumor necrosis and trauma, and coagulopathy would lead to hemorrhage within the tumor. These mechanisms would further increase the pressure in the HCC tumor, resulting in the splitting of the overlying liver parenchyma and the rupture of the HCC into the liver surface. The results obtained in our study were consistent with this hypothesis proposed by Zhu et al. In all the patients in our study, the ruptured HCCs were located on the surface of the liver. We speculated that the risk of hepatic vein invasion increases with increasing HCC tumor size. Therefore, the risk of rupture also increases with increasing tumor size for tumors located on the surface of the liver. Furthermore, TAE remains unsatisfactory in patients with large tumors, and further improvements in the prognosis of patients with large tumors on the surface of their livers may be difficult to obtain using TAE.

## Conclusion

Spontaneous rupture is a serious complication of HCC, and the 30-day survival in the absence of any treatment is extremely low. The early mortality rate among patients with ruptured HCC depends on the immediate treatment provided. TAE is often feasible and is the treatment of choice for ruptured HCC. Despite the limitations of this retrospective study, we preferentially utilized TAE to stop bleeding in the management of ruptured HCC. Among cases where initial TAE is successfully performed, the size of the tumor is a very important factor influencing the survival of cirrhotic patients with ruptured HCC.

## Competing interests

The authors declare that they have no competing interests.

## Authors' contributions

HK and SS performed the literature review, collected the clinical data, and drafted the manuscript, with contributions from MY, KF, HM, TU and TH. SK and YN organized the field survey for data collection. KI, TS, KA, AS, AK, HT, YA, MI, NK, KK and NU collected the clinical data. SS and AN were responsible for the design of the study. All authors read and approved the final manuscript.

## Pre-publication history

The pre-publication history for this paper can be accessed here:

http://www.biomedcentral.com/1471-230X/9/29/prepub
